# Gallic acid nanoflower immobilized membrane with peroxidase-like activity for m-cresol detection

**DOI:** 10.1038/s41598-020-73778-7

**Published:** 2020-10-07

**Authors:** Seyma Dadi, Cagla Celik, Ismail Ocsoy

**Affiliations:** Department of Analytical Chemistry, Faculty of Pharmacy, Erciyes University, 38039 Kayseri, Turkey

**Keywords:** Biotechnology, Chemistry, Materials science, Nanoscience and technology

## Abstract

We report fabrication of new generation nanoflowers (NFs) using gallic acid (GA) and copper (II) ions (Cu^2+^) acted as an organic and inorganic component, respectively with effective peroxidase mimic activities in solution and on filter membrane. Unlike the typical protein NFs synthesis mechanism, gallic acid NFs (GA-NFs) was formed via coordination reaction between carboxyl groups of GA and Cu^2+^. The different morphologies of the GA-NFs were acquired based upon whether the carboxyl groups in gallic acid are active or not. The peroxidase mimic activity of the GA-NFs relied on the Fenton reaction in the presence of hydrogen peroxide (H_2_O_2_) was tested towards m-cresol as a function of concentration of the GA-NFs, m-cresol, H_2_O_2_ and reaction time. Under the optimized conditions, the oxidative coupling of m-cresol with 4-aminoantipyrine (4-AAP) was catalyzed by the GA-NFs dispersed in solution and adsorbed on filter paper to form an antipyrine dye and it was visually and spectrophotometrically recorded. The m-cresol with range of 0.05–0.5 mM was detected in 10 min and 15 min by using the GA-NFs in solution and on filter paper, respectively. We demonstrated that the NFs can be produced from non-protein molecules and GA-NFs can be used as a promising nanocatalyst for a variety of applications.

## Introduction

Recently, enzyme-inorganic flower-shaped hybrid nanostructures called “nanoflower (NF)” have received considerable attention owing to their greatly enhanced catalytic activity and stability compared to free and conventionally immobilized enzymes. Reasons for elevated activity and stability of the enzyme NFs can be: (1) high surface area, (2) alleviated mass-transfer limitations, (3) the increase in local concentration of the enzymes and (4) morphology dependent cooperative effect. The typical formation mechanism of proteins/enzymes-based NFs relies on forming complexes between amide groups of proteins/enzymes backbone and Cu^2+^ ions in phosphate buffer solution (PBS). Initially, Cu^2+^ ions reacted with phosphate ions (PO_4_^3−^) to form Cu_3_(PO_4_)_2_ primary crystals, then, protein molecules bind to Cu_3_(PO_4_)_2_ primary crystals through coordination reaction between amide group and Cu^2+^ to from protein-Cu_3_(PO_4_)_2_ complexes as seeds, which can be called “ nucleation step”. The continuous feeding of Cu_3_(PO_4_)_2_ with protein results in seed growth for formation large petals and these petals are combined each other leading to multi-branched flower like structures via anisotropic growth, which can be called “growth step”^1–6^. Finally, the growth of nanoflowers reaches to saturation and their morphology is completed, which can be called “completion step”. Up to now, various enzymes-based NFs have been produced and utilized in many bioanalytical applications^7–12^. For instance, Wang and coworkers reported the synthesis of glucose oxidase (GOx) and horseradish peroxidase (HRP)-Cu_3_(PO_4_)_2_ hybrid NF for detection of glucose as a colorimetric sensor^9^. Additionally, Lu and coworkers developed a portable test kit prepared with acetylcholinesterase-NF incorporated agarose hydrogel for visual detection of acetylcholine^10^.

Moreover, while DNA capped gold or iron oxide nanoparticles (NPs) exhibited intrinsic peroxidase-like activity as DNA and colloidal NPs combination^13,14^, recent studies have shown that the amino acids, catecholamine and plant extracts can act as organic components and Cu^2+^ ions used as inorganic components for fabrication of the NFs instead using proteins/enzymes as organic molecules^12,15,16^. It is worthy to mention that amine and/or carboxyl groups in biological or organic molecules also coordinately react with Cu^2+^ ions to build up non-protein NFs. They have exhibited peroxidase like and antimicrobial activities benefiting from Fenton reaction mechanism in the presence of hydrogen peroxide (H_2_O_2_). However, no synthesis of NFs using amine group free molecules has been reported and formation mechanism has not been documented yet.

Phenol and its derivatives are industrial by product used in many processes, such as the production of pesticides, insecticides, plastics and dyes and have serious negative effects to human and to the environment^17^. Therefore, the development of analytical methods for determination of these compounds is of great importance. The several approaches or methods have been developed for analyzing these compounds including chromatographic^18^ and electrochemical methods^19^. However, these methods require expensive instruments, multi-step and time-consuming procedures and trained personnel as well. Colorimetric sensing has drawn attention due to low cost, easy to operate, one-step and rapid procedure for detection of phenolic compounds^20^.

Herein, we report, for the first time, the synthesis of gallic acid-based NFs (GA-NFs) with effective peroxidase mimic catalytic activity for colorimetric and spectrophotometric detection of m-cresol. The GA-NFs exhibited peroxidase mimic activity in the presence of H_2_O_2_ based on Fenton reaction. Basically, GA-NFs catalyzed oxidation of series of concentrations (0.05 mM, 0.1 mM, 0.2 mM, 0.3 mM, 0.4 mM, 0.5 mM) of m-cresol with of 4-AAP in solution within 10 minute (min) incubation and the products were spectrophotometrically and visually detected. Additionally, the same procedure was applied to GA-NFs immobilized filter membrane, then the detection of m-cresol with same concentrations were accomplished in 15 min. Various parameters such as concentration of GA-NFs, H_2_O_2_ and reaction time were investigated to optimize the detection of m-cresol in solution and on filter membrane.

## Results

### Synthesis of GA-NFs

The synthesis of organic–inorganic nanoflowers (NFs) relies on coordination reaction between amide groups in the protein backbone and Cu^2+^ in Cu_3_(PO_4_)_2_ primary crystals. Although, non-protein molecules including amino acids (known as the building blocks of proteins), catecholamines or some model plants extracts acted as organic components of the NFs, almost all recent studies have taken the coordination between the amide group and Cu^2+^ as a key step for formation of the NFs. It seems that the selection of amide group containing molecules have become a mandatory in NFs synthesis, which can be considered as a major disadvantage of the NFs formation.

**Scheme 1:**
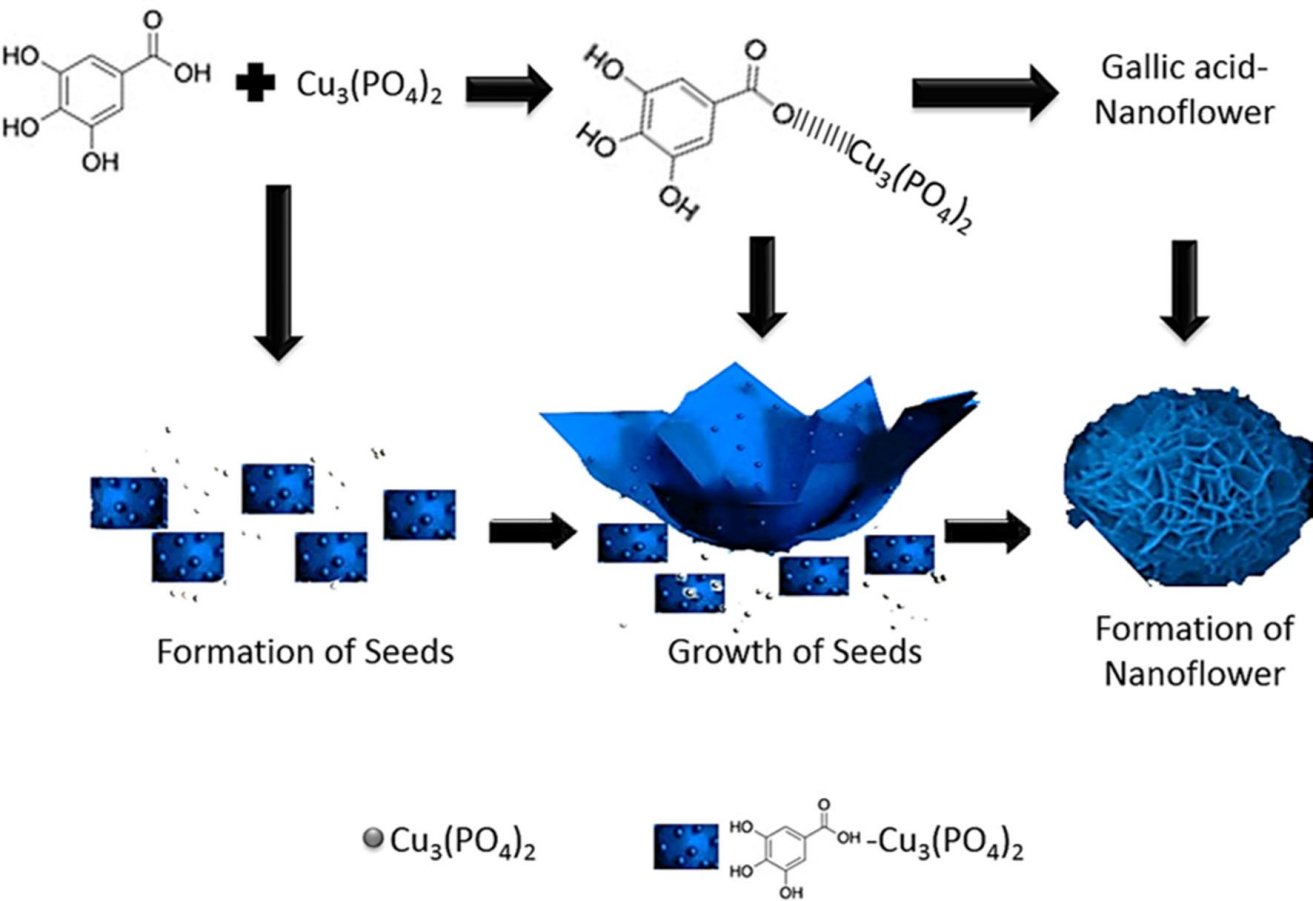
Illustration of potential formation mechanism of GA-NFs with three successive: formation of seed, growth of seed and formation of nanoflower. *Note*: Grey dot represents Cu_3_(PO_4_)_2_ primary crystals and Grey dot incorporated blue rectangular represents GA-Cu_3_(PO_4_)_2_ complex.

Herein, we present, for the first time, an inspirational work with fabrication of gallic acid incorporated nanoflower (GA-NFs) by exploiting coordination reaction occurred between carboxyl group of GA and Cu^2+^. The typical NFs synthesis procedure was applied that free GA was added into 10 mM PBS solution containing Cu^2+^, and then the resulting mixture was left for incubation without disturbing. The carboxyl group of GA reacted with Cu^2+^ in Cu_3_(PO_4_)_2_ primary crystals to initiate the formation of GA-Cu_3_(PO_4_)_2_ complexes as seeds. Interestingly, GA-Cu_3_(PO_4_)_2_ complexes were grown as large petals and these GA-incorporated petals bound to each other by acting as a glue, as protein incorporated ones acted in discovery of NFs before. Then, complete flower-shaped structure called “nanoflower (NF)” is occurred with saturation of anisotropic growth. The formation mechanism of GA-NFs was demonstrated in Scheme 1 step by step.

As an interesting and worthy approach, the roles of activation of carboxyl group on the morphologies of GA-NFs were systematically examined. The 1-Ethyl-3-(3-dimethylaminopropyl) carbodiimide (EDC) and N-hydroxysuccinimide (NHS) in standard protein labeling chemistry or called “EDC/NHS chemistry” was utilized to activate the carboxyl group of free GA. Then, typical NF synthesis procedure was followed to show how activated carboxyl group influence morphology of GA-NFs.

**Figure 1:**
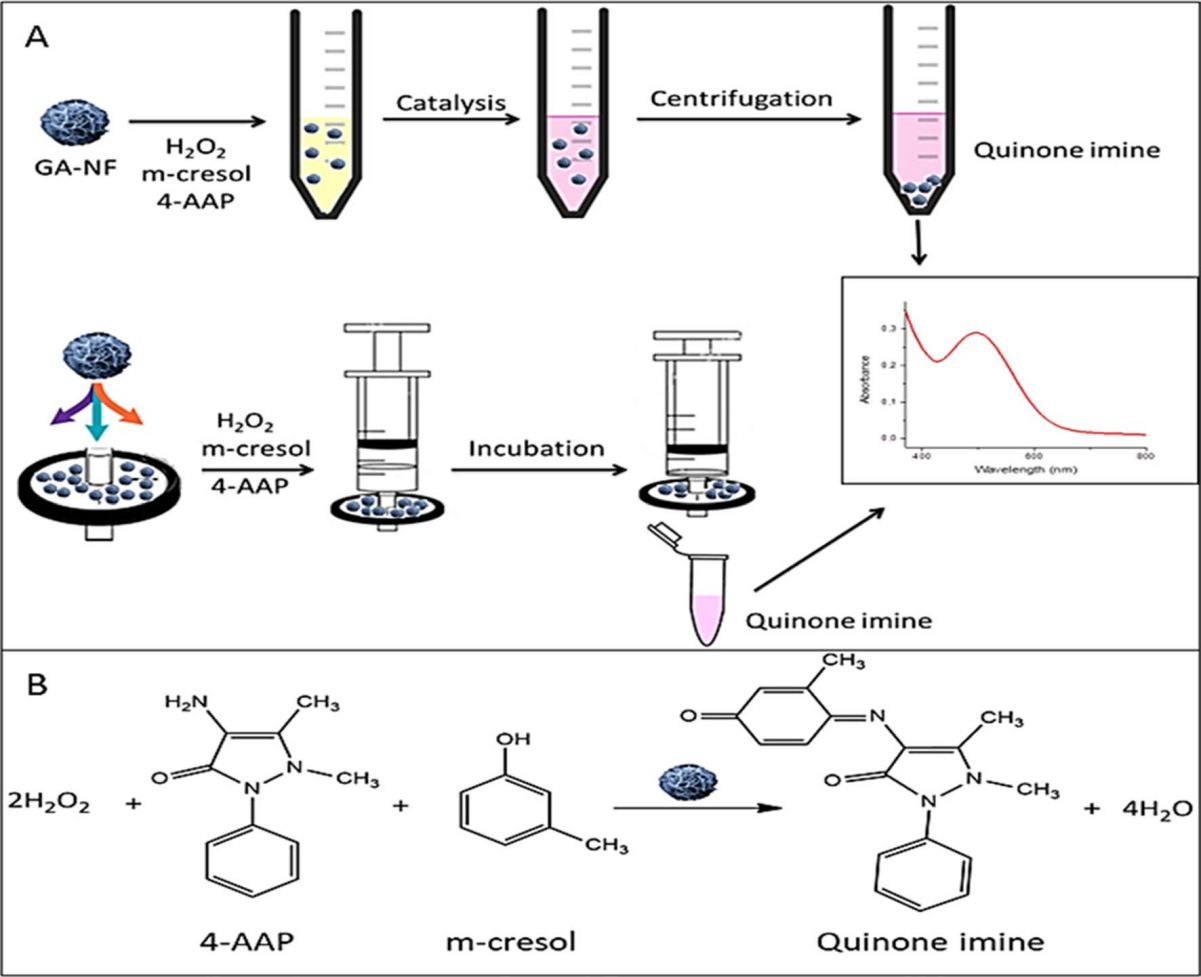
Schematic illustration of (**A**) peroxidase activities of the cGA-NFs dispersed in solution and adsorbed on filter membrane and (**B**) cGA-NFs catalyzed oxidative coupling between m-cresol and 4-AAP for formation of a colored antipyrine dye.

Structure of the GA-NFs with peroxidase mimic activities in solution and on filter membrane were characterized and interpreted. In our system, the GA-NFs were dispersed in solution and physically adsorbed on filter membrane for detection of m-cresol known as important hazardous compound, by UV–Vis spectrophotometer and naked eye (Fig. 1A). The oxidative coupling reaction between m-cresol and 4-AAP catalyzed by GA-NFs in the presence of H_2_O_2_ was shown in Fig. 1B.

### Characterization of GA-NFs

**Figure 2:**
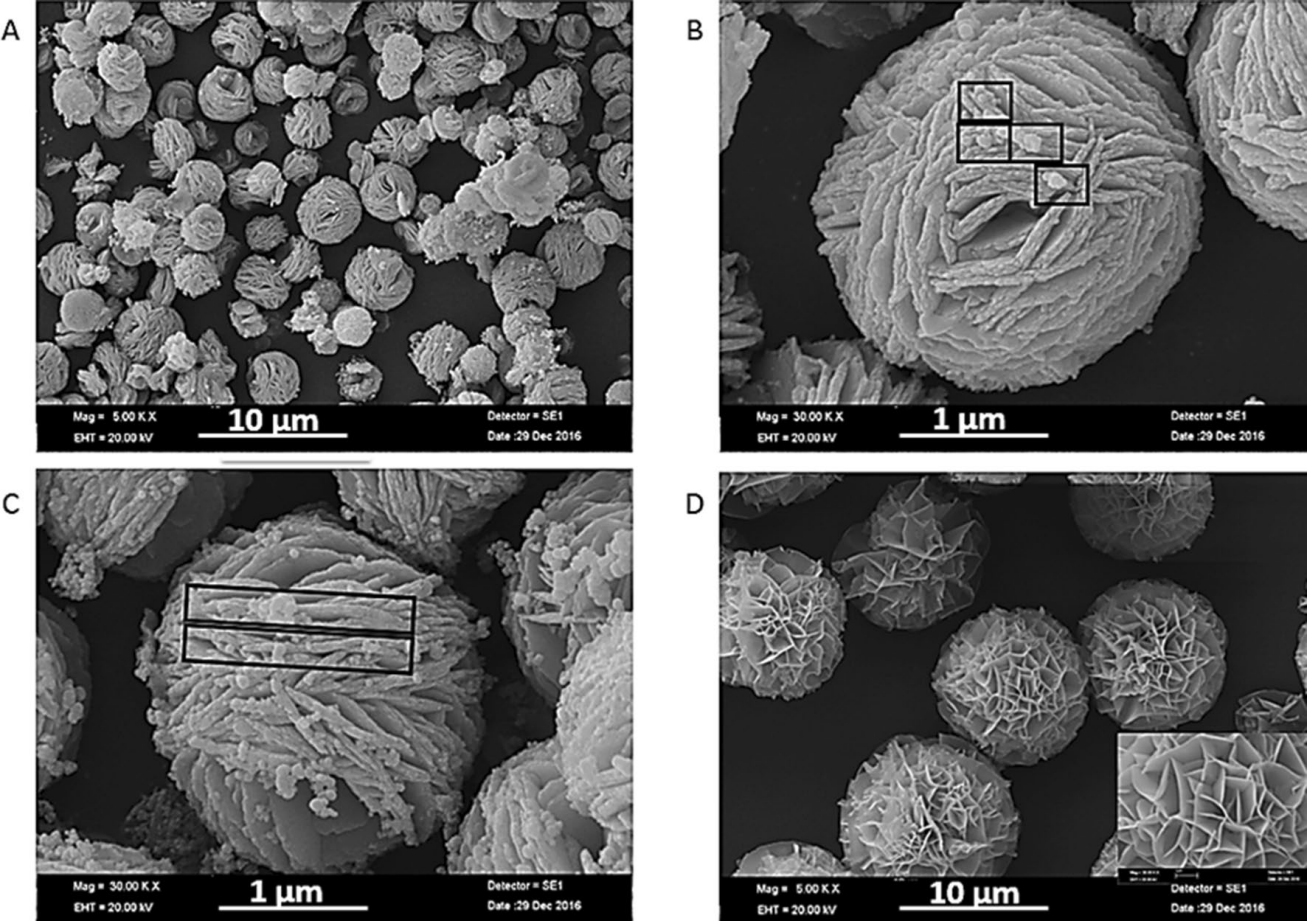
Images of the GA-NFs formed in (**A**, **B**) 3 days, (**C**) 4 days and (**D**) image of the carboxyl group activated cGA-NFs formed in 3 days. Inset: high-magnification image of Fig. 2D.

The structure of GA-NFs was characterized via several methods. The morphologies (shape, size and surface property) of GA-NFs and carboxyl group activated GA-NFs (cGA-NFs) were monitored by scanning electron microscopy (SEM). The SEM images in Fig. 2A, B show that GA-NFs are spherical with ~ 4 µm size. It seems that the GA-incorporated large petals have plate like shapes, and they have vertically inserted each other to form the GA-NFs. The small spheres (shown in black square) on surface of magnified GA-NFs image in Fig. 2B can be indication of newly occurred GA-Cu_3_(PO_4_)_2_ nanocrystals. We hypothesize that formation of the GA-NFs may be kinetically slow and under continuous formation process. Figure 2C shows that when reaction time was prolonged, the small spheres grew and wrapped surface of the GA-NFs as belts (shown in black rectangular). With the worthy approach, we activated carboxyl group of GA to facilitate coordination reaction between GA and Cu^2+^. And then, the ~ 9 µm sized, uniform, and mono-dispersed cGA-NFs with highly porous structure were produced as shown in Fig. 2D. The high-magnification image of the cGA-NFs was also presented in inset of Fig. 2D.

**Figure 3:**
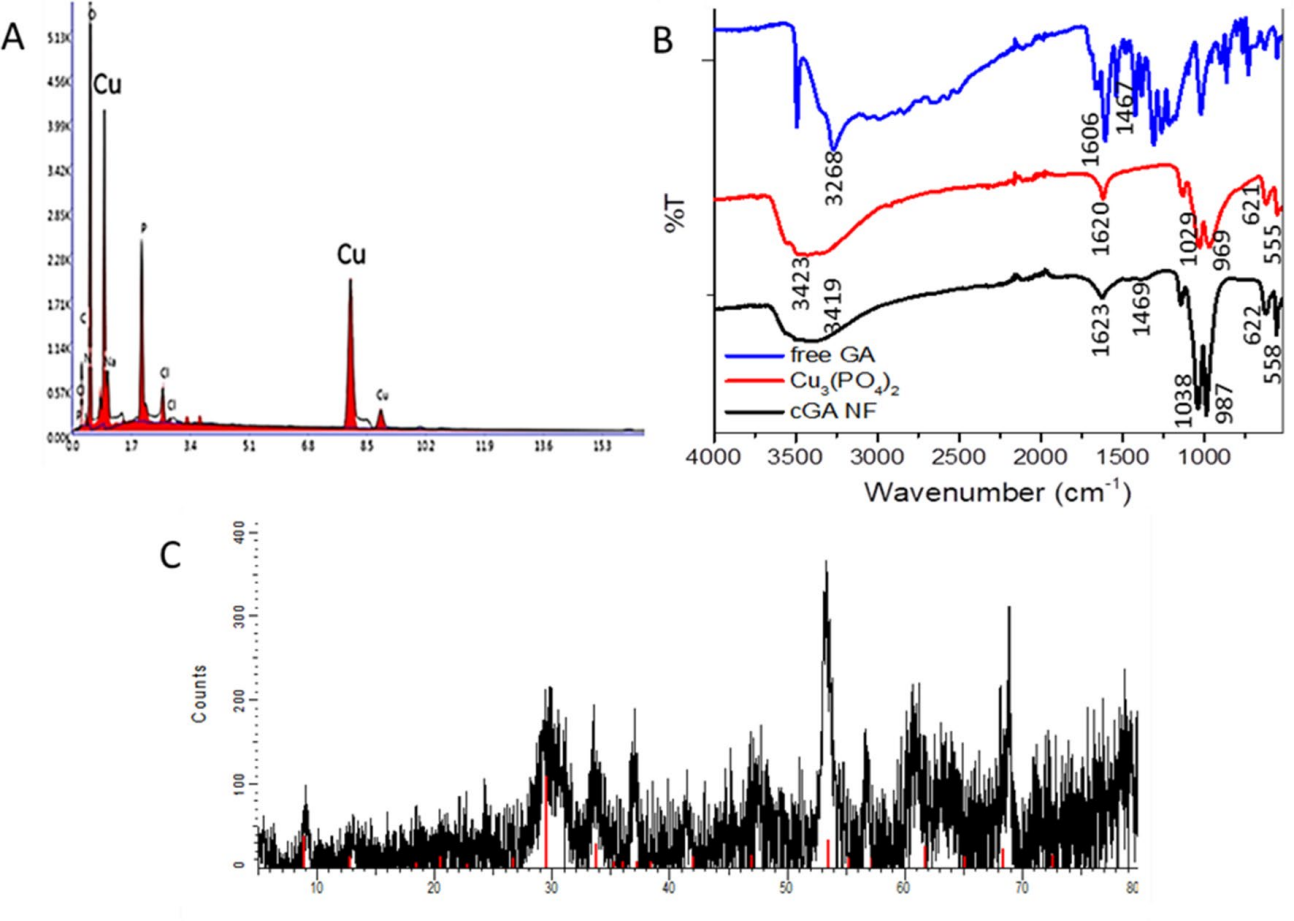
The structural analysis of cGA-NFs. (**A**) EDX analysis for determination of Cu metal, (**B**) FT-IR spectrum for evaluation of characteristic peaks and (**C**) XRD pattern of cGA-NFs (black line). Peak position of the Cu_3_(PO_4_)_2_·3H_2_O (red line, JPSCD 00-022-0548).

As a further structural analysis, Energy Dispersive X-ray Analysis (EDX) was used to show the presence of Cu metal in the Cu_3_(PO_4_)_2_ scaffold (Fig. 3A). As the formation mechanism of the NF have been well documented, metal ions, especially Cu^2+^ acts as an indispensable cornerstone component for building of the NF. The bending and stretching in the cGA-NFs were evaluated with Fourier-transform infrared spectroscopy (FTIR) as shown in Fig. 3B. For analysis of free GA (blue line), the stretching of O–H groups at 3268 cm^-1^, strong absorption of COOH (carboxylic acids) at 1606 cm^-1^ and C=O stretching at 1467 cm^-1^ are attributed to characteristic peaks of free GA. The characteristic peaks of 1029 cm^-1^ and 555 cm^-1^ refer to PO_4_^3−^ vibrations of Cu_3_(PO_4_)_2_ as given with red line^15^. While the PO_4_^3−^ vibrations in cGA-NFs are assigned to 1038 cm^-1^ and 558 cm^-1^, the stretching of O–H group with different mode, moderate absorption of COOH and C=O stretching are observed at 3419 cm^-1^, 1623 cm^-1^, 1469 cm^-1^, respectively. The consistency in the FTIR spectra is an indication of cGA incorporated NFs. The diffraction peak positions of cGA-NFs spectrum show that the crystal pattern of Cu_3_(PO_4_)_2_·3H_2_O (JPSCD 00–022-0548) and NaCl (JPSCD 01-088-2300) can be both seen in XRD spectrum (Fig. 3C).

### Detection of m-cresol

The m-cresol and its s isomers o-cresol and p-cresol can be enzymatically oxidized but with different efficiencies owing to favorable position of ring substituents in their structures^21,22^. We chose m-cresol for catalytic reaction due to high substrate specificity at room temperature (RT: 20 °C). After the synthesis of cGA-NFs, we demonstrated carboxyl group activated cGA-NFs exhibited much enhanced peroxidase mimic activity compared to GA-NFs formed of GA molecule containing non-activate carboxyl group. The reasons for morphology dependent activity can be attributed to highly porous and compact structures of cGA-NFs. As shown in Figure S1A, the cGA-NFs display higher peroxidase like activity to catalyze the reaction between 4-AAP and m-cresol in the presence of H_2_O_2_ than GA-NFs. Then, we only used cGA-NFs in all peroxidase mimic activity experiments. The cGA-NFs acted as a Fenton reagent in the presence of H_2_O_2_ and then exhibited peroxidase mimic activity through Fenton reaction. The potential mechanism for Fenton reaction is that Cu^2+^ ions in the cGA-NFs react with H_2_O_2_ to produce Cu^1+^. Followingly, interaction between Cu^1+^ and H_2_O_2_ resulted in highly reactive hydroxyl radical (·OH), which catalyzes oxidative coupling reaction between m-cresol and 4-AAP to form an antipyrine dye as a colored compound. The mechanism for the potential Fenton-like reaction is given in Eq. (1).

1 $$\begin{gathered} {\text{Cu}}^{{2 + }} + {\text{H}}_{{2}} {\text{O}}_{{2}} \to {\text{Cu}}^{ + } + {\text{HOO}}^{ \cdot } + {\text{H}}^{ + } \hfill \\ {\text{Cu}}^{ + } + {\text{H}}_{{2}} {\text{O}}_{{2}} \to {\text{Cu}}^{{2 + }} + {\text{HO}}^{ \cdot } + {\text{OH}}^{ - } \hfill \\ \end{gathered}$$

**Figure 4:**
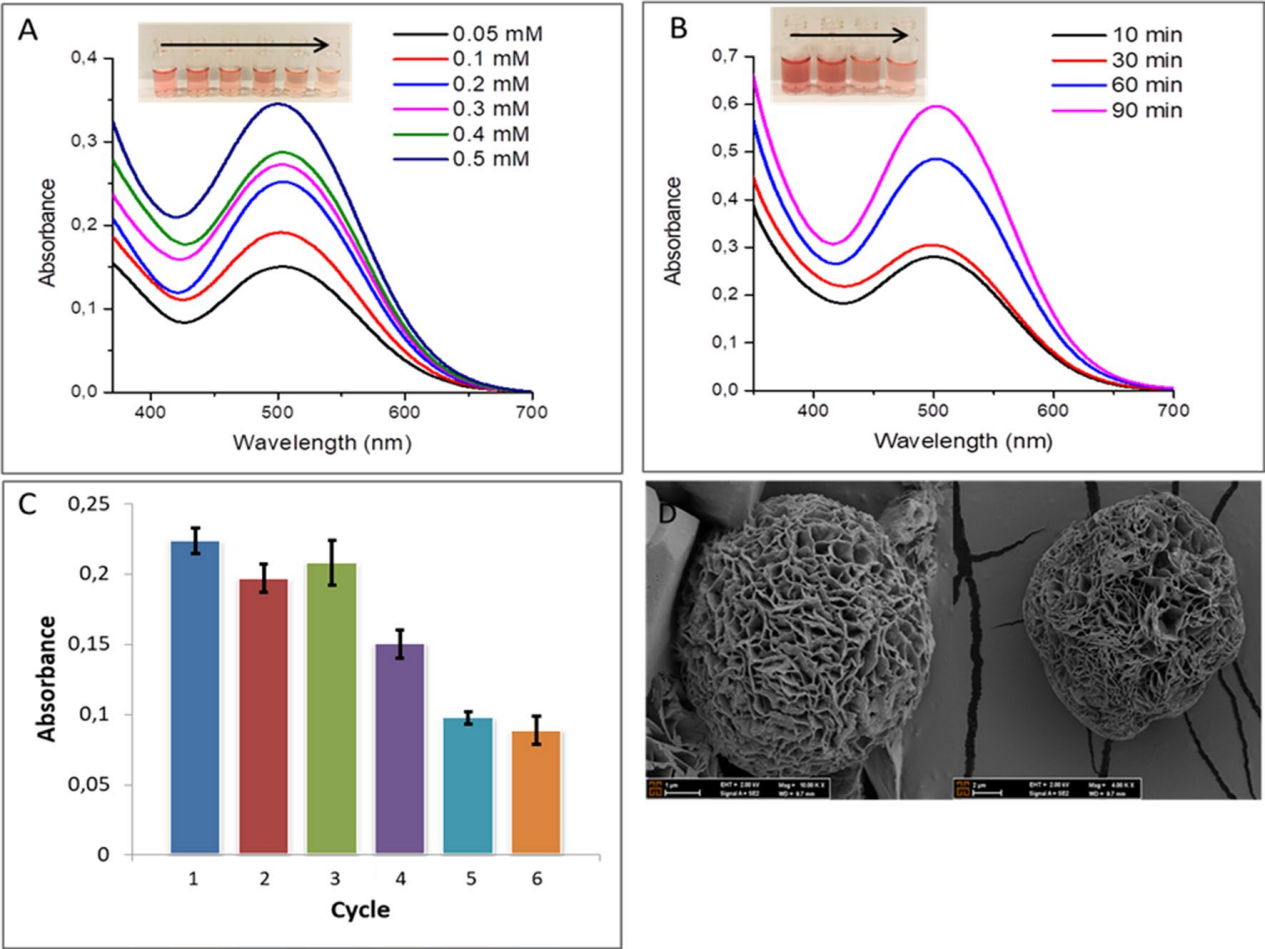
Peroxidase mimic activities of the cGA-NFs dispersed in solution as a function of (**A**) m-cresol concentration, (**B**) reaction time, (**C**) repeated use (**D**) SEM image of cGA-NFs before reaction and after six cycle reaction.

The peroxidase mimic activities of the cGA-NFs were evaluated in solution and on filter membrane against m-cresol under the various experimental parameters. In Fig. 4, the peroxidase mimic activity of the cGA-NFs dispersed in solution was studied. A standard activity protocol was followed; oxidative coupling between m-cresol and 4-AAP was catalyzed by the cGA-NFs with intrinsic peroxidase-mimic activity. The cGA-NFs (0.5 mg/mL) was added into solutions containing 4 mM 4-AAP, 1 mM H_2_O_2_ and m-cresol with a series of concentrations (0.05 mM, 0.1 mM, 0.2 mM, 0.3 mM, 0.4 mM, 0.5 mM) and each solution was incubated for 10 min, then its activity was tested in each solution as a function of m-cresol concentrations (Fig. 4A). It reveals that absorption values and color intensity (the direction of the arrow in the photo is from high concentration to low in Fig. 4A) of the product solutions were increased with increase in m-cresol concentrations. The same activity protocol in Fig. 4A was used; activity of the cGA-NFs was evaluated towards m-cresol fixed to 0.4 mM for different incubation times. 0.4 mM m-cresol, the cGA-NFs (0.5 mg/mL), 4 mM 4-AAP and 1 mM H_2_O_2_ were mixed together and incubated for 10 min, 30 min, 60 min and 90 min. The absorbance of each solution was measured by UV–Vis. Figure 4B shows that although intense color change and high absorbance values after catalytic reactions was obtained in long incubation times (90 min and 60 min), in 10 min or even in 5 min (data now shown) the remarkable color change and absorption increase were observed (the direction of the arrow in the photo is from high incubation time to short in Fig. 4B). The effect of presence of H_2_O_2_ was shown in Supplementary Information Figure S2. It reveals that the efficiency of oxidative coupling reaction between 4-AAP and m-cresol was enhanced with H_2_O_2_. To demonstrate the activity and stability of the cGA-NFs, the reaction solution containing 0.5 mg/mL cGA-NFs, 0.4 mM m-cresol, 4-AAP and 1 mM H_2_O_2_, was applied successive catalytic use (Fig. 4C). We clearly showed that the cGA-NFs lost 60% its initial catalytic activity over the six cycles, which may exhibit high catalytic performance and stability. SEM images of the GA-NFs were recorded before reaction (left one in Fig. 4D) and after the six cycles (right one in Fig. 4D). It is clear that the morphology of the GA-NFs was slightly distorted after six cycles in use compared to intact cGA-NFs image.

**Figure 5:**
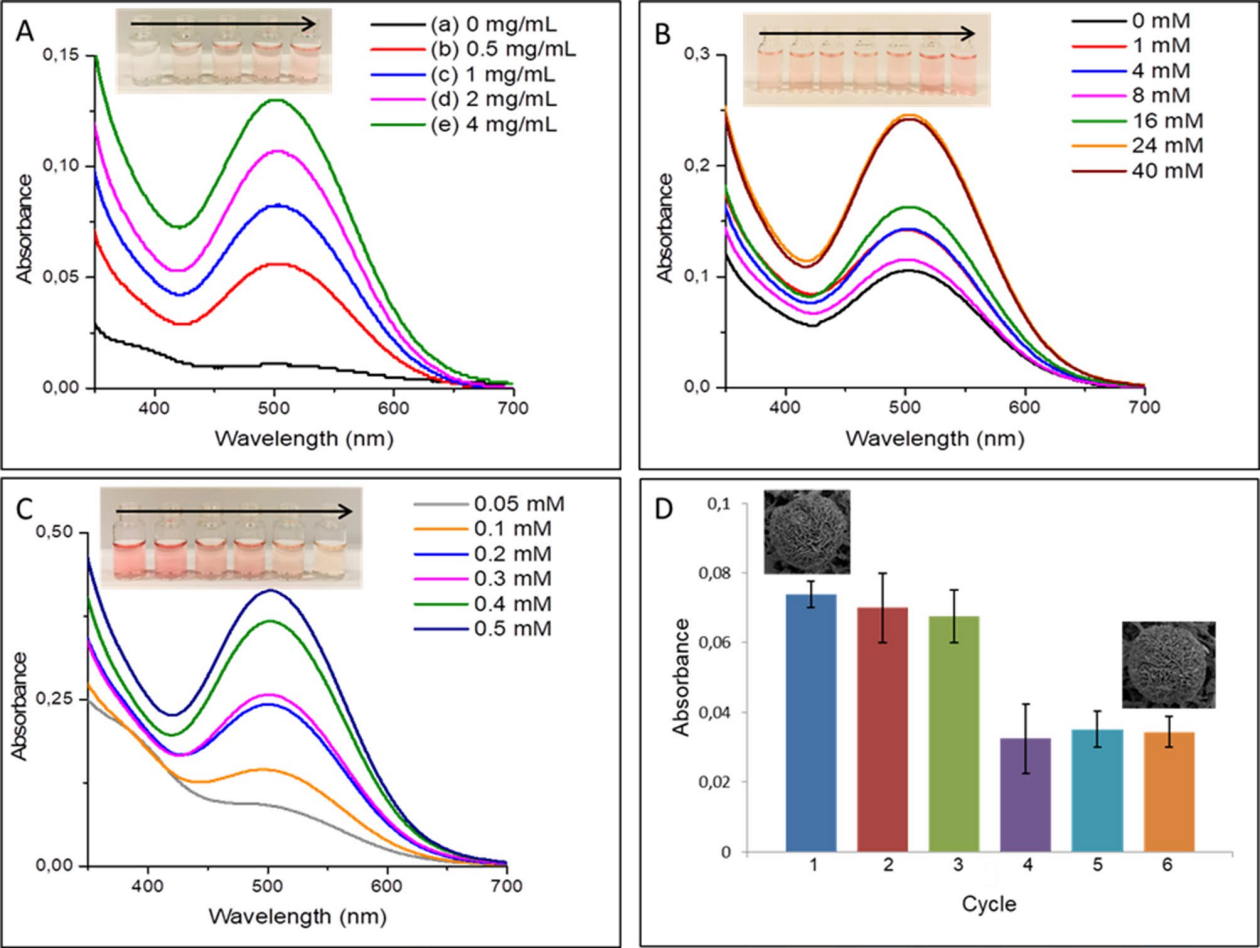
Peroxidase mimic activities of the cGA-NFs adsorbed on filter membrane as a function of (**A)** NFs concentration, (**B**) H_2_O_2_ concentration, (**C**) m-cresol concentration, (**D**) repeated use.

To use the cGA-NFs as an attractive nanobiocatalyst, we non-covalently deposited the cGA-NFs on the surface of commercial filter membrane, then investigate how it exhibit peroxidase mimic activity as function the cGA-NFs, m-cresol and H_2_O_2_ concentration and reaction time. As a first parameter (Fig. 5A), a series concentration of the cGA-NFs was absorbed on filter membranes, then reaction solution (4 mM 4-AAP, 1 mM H_2_O_2_ and 0.4 mM m-cresol) was injected each membrane to observe its activity in 15 min. It is noticed that using the filter membrane high amount of the cGA-NFs exhibited much efficient catalytic activity, which is quite consistent with absorption value and color intensity of the product solution (the direction of the arrow in the photo is from low amount cGA-NFs to high on filter membrane in Fig. 5A). We realized that 2 mg/mL cGA-NFs adsorbed filter membrane can be ideal nanobiocatalyst for further reaction owing to effective peroxidase mimic activity. As aforementioned above, presence of H_2_O_2_ and its concentration are vitally important for rapid and efficient catalytic activity as shown in Fig. 5B. We fixed the concentration of the cGA-NFs on filter membrane, m-cresol and 4-AAP to be 2 mg/mL, 0.4 mM and 4 mM, respectively, then concentrations of H_2_O_2_ were varied. Although we do not expect any catalytic activity in the absence of H_2_O_2_, the oxidation of m-cresol was carried out catalyzed by the cGA-NFs adsorbed filter membrane as demonstrated with absorption value and color change. We hypothesize that Cu^2+^ ion in the cGA-NFs react with m-cresol to give complexation reaction for formation of a chelate, then Cu^2+^ is reduced to Cu^1+^and oxidative coupling reaction of m-cresol with 4-AAP is occurred under the redox reaction. Interestingly, the filter membrane exhibited the highest catalytic activity in both 40 mM and 24 mM H_2_O_2_ as absorption values and color intensity of the product solution dictate this phenomenon (the direction of the arrow in the photo is from low concentration of H_2_O_2_ to high in Fig. 5B). After determining the ideal concentration of the cGA-NFs and H_2_O_2_, the effect of the m-cresol concentration was examined. Figure 5C shows that while even lowest concentration of m-cresol (0.05 mM) was oxidized by the filter membrane and spectrophotometrically and visually detected but in 60 min, however, almost the same catalytic performance was obtained when using 0.5 mM and 0.4 mM m-cresol. The recycling of the filter membrane was tested over the six catalytic cycles, the filter membrane maintained almost 60% of its first cycle activity even after six cycles (Fig. 5D). We hypothesize that favorable conformation of the cGA-NFs can be slightly changed after third cycle was, then gradual reduction in catalytic activities were observed after third cycle. And, the distance between petals or layers of cGA-NFs can decrease and they may stick each other, then catalytic activity can be adversely influenced after third cycle wash. We claim that the cGA-NFs adsorbed on filter membrane possessed much durability compared to the cGA-NFs dispersed in solution, as how the enzyme NFs show enhanced stability compared to free enzymes. We also monitored how successive catalytic reactions influence the morphologies of the cGA-NF on filter membrane with SEM images. The SEM image of the filter membrane were obtained after first and six cycles as seen on top of blue column (image after first cycle) and orange column (image after six cycles), we analyzed that no remarkable distortion on both SEM images. The potential reasons for reduction in catalytic activity of the filter membrane after repeated use can be i)adsorption of excess m-cresol on surface of the cGA-NFs may increase mass-transfer limitations and ii) excess m-cresol may attack available or accessible Cu^2+^ ions and form m-cresol-Cu^2+^ complexes, both of which may obstruct catalytic activity performance of the NFs. it is worthy to point out that although the morphology of the NFs is not significantly altered or impaired, the catalytic activity decreases due to the reasons mentioned above.

## Discussion

In summary, we have developed cGA-NFs as new generation NFs and investigate their peroxidase mimic activities through the Fenton reaction when dispersed in solution and adsorbed on filter membrane towards m-cresol. In synthesis procedure, carboxyl group of GA molecules reacted with Cu^2+^ for formation of cGA-Cu_3_(PO_4_)_2_ primary nanocrystals, then cGA-NFs was kinetically formed. We also activated carboxyl group of GA, then we showed how it influences morphology and peroxidase mimic activity of the cGA-NFs. The peroxidase-mimic activities of the cGA-NFs in solution and on filter membrane was optimized under various experimental parameters. The both synthesis of the cGA-NFs and the preparation of cGA-NFs deposited filter membrane opened up new avenue in designing novel biocatalytic system. The uniform, mono-dispersed and porous cGA-NFs with intrinsic peroxidase-mimic activity can be promising alternative to enzyme-incorporated NFs and find widespread use in various scientific and technical fields.

## Materials

Copper(II) sulfate pentahydrate (CuSO_4_·5H_2_O), m-cresol, gallic acid, 4-Aminoantipyrine (4-AAP), hydrogen peroxide (H_2_O_2_, 25% w/v) , salt precursor of PBS (NaCl, KCl, Na_2_HPO_4_, KH_2_PO_4_, CaCl_2_.2H_2_O, MgCl_2_.6H_2_O), 1-Ethyl-3-(3-dimethylaminopropyl) carbodiimide (EDC) and N-hydroxysuccinimide (NHS) were purchased from Sigma Aldrich. Cellulose acetate syringe filters (0.45 µm) were obtained from Isolab. All solutions were prepared with ultrapure water (resistance 18.2 MΩ). All chemicals were of analytical grade and used without further purification.

## Methods

UV–Vis spectrophotometry (Shimadzu UV1800) was used for determination of peroxidase like activity of NFs. Scanning Electron Microscopy (SEM, ZEISS EVO LS10) was operated for imaging morphologies of NFs. IR spectra of NFs was recorded on a FT-IR (Thermo Scientific Nicolet 6700). Crystal structure of NFs and Cu_3_(PO_4_)_2_ primary crystal were analyzed by X-Ray Diffraction (XRD, Bruker AXS D8 Advance Model).

### Formation of gallic acid nanoflowers

Organic inorganic nanoflowers were synthesized according to literature with some modifications^16^.Gallic acid was used as an organic part and Cu^2+^ ion acted as an inorganic part for the synthesis of GA-NF. Briefly, 0.02 mg/mL gallic acid was dissolved in distilled water. An aqueous solution of CuSO_4_ (120 mM, 660 µL) and gallic acid was added to 100 mL phosphate buffer saline (PBS) solution (10 mM, pH 7,4). The mixture was stirred vigorously for 5 min to increase interaction between Cu^2+^ and gallic acid. After the incubation at 25 °C for 3 days without disturbing, the precipitates formed at the bottom of the solution were collected through centrifugation (5000 rpm, 10 min) and washed with pure water several times. The obtained product was dried at 50 °C.

For the synthesis of cGA-NFs, 0.02 mg/mL was dissolved in PBS solution (10 mM, pH 7.4). The solution was mixed with EDC (10 mM) and NHS (12 mM), following by stirring overnight at room temperature. Then an aqueous solution of CuSO_4_ (120 mM, 660 µL) was added to mixture and incubated at 25 °C for 3 days. The obtained products bottom of the solution were collected through centrifugation (5000 rpm, 10 min) and washed with pure water several times. The cGA-NFs was dried at 50 °C.

### Detection of m-cresol in solution

Different concentration of m-cresol (0.05 mM, 0.1 mM, 0.2 mM, 0.3 mM, 0.4 mM and 0.5 mM) was added to PBS solution (0,1 M pH 7,4) containing cGA-NFs (1 mg/mL), H_2_O_2_ and 4-AAP (4 mM). These solutions were incubated at room temperature for 15 min, followed by centrifugation at 8000 rpm for 5 min to remove cGA-NFs. The absorbance of resulting solutions was measured by UV–Vis spectrophotometer.

### Detection of m-cresol on filter membrane

The GA-NF suspension in PBS was deposited on filter membrane (cellulose acetate membrane 0.45 µm) and air was pressed through the filter to remove PBS. Subsequently, the mixture containing different concentration of m-cresol, 4 mM 4-AAP and H_2_O_2_ was injected to filter with syringe. After incubation at room temperature for various periods of times on filter membrane, the product was collected into glass vial and the absorbance of the product was recorded by UV–Vis spectrophotometer.

## Supplementary information

Supplementary file1
