# Supplementary material for: Gallic acid nanoflower immobilized membrane with peroxidase-like activity for m-cresol detection
Source: Sci Rep. 2020 Oct 7;10:16765. doi: 10.1038/s41598-020-73778-7 (PMC7542149; doi:10.1038/s41598-020-73778-7)
Supplement: Supplementary file 1 — Supplementary file1 [file 41598_2020_73778_MOESM1_ESM.docx]

**Gallic acid nanoflower membrane with peroxidase-like activity for m-cresol  detection**

Seyma Dadi^1^, Cagla Celik^1^ and Ismail Ocsoy^1*^

^1^Department of Analytical Chemistry, Faculty of Pharmacy, Erciyes University, 38039, Kayseri, Turkey


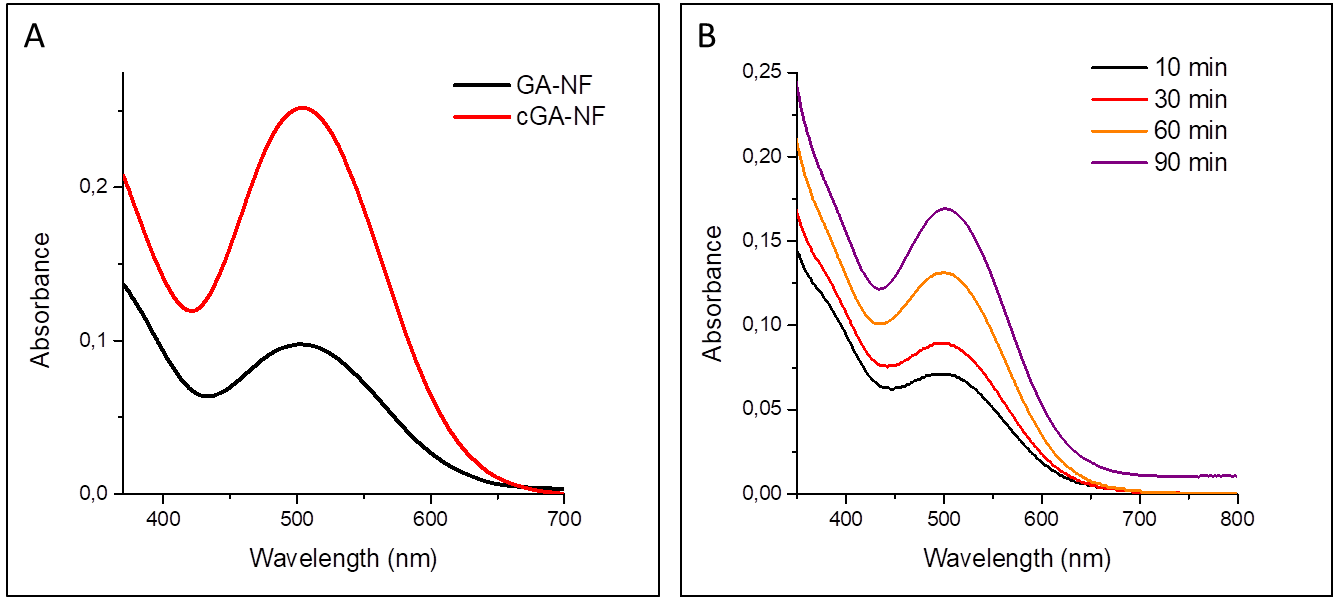


**Fig. S1.** A) Peroxidase mimic activities of the GA-NFs and cGA-NFs dispersed in solution B) Peroxidase mimic activities of the GA-NFs dispersed in solution as a function of reaction time.


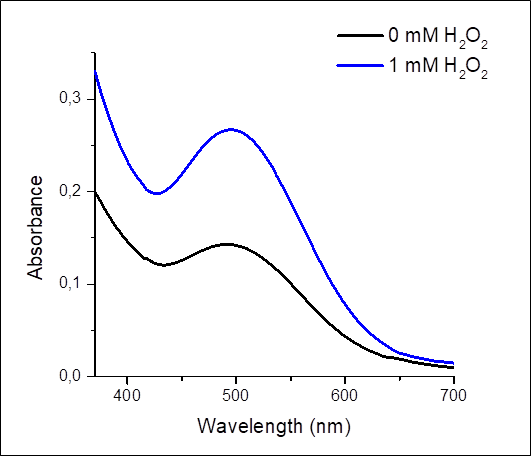


**Fig. S2.** Peroxidase mimic activities of the cGA-NF dispersed in solution as a function of H_2_O_2_ concentration


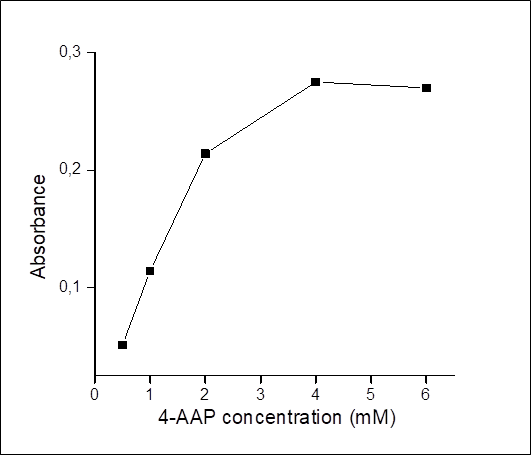


**Fig. S3.** The effect of 4-AAP concentration in solution in the presence of 0.5 mg/mL cGA-NFs, 0.4 mM m-cresol, 1 mM H_2_O_2_.
